# Further Association of Germline *CHEK2* Loss-of-Function Variants with Testicular Germ Cell Tumors

**DOI:** 10.3390/jcm12227065

**Published:** 2023-11-13

**Authors:** Kira Kirchner, Christoph Seidel, Finn-Ole Paulsen, Bianca Sievers, Carsten Bokemeyer, Davor Lessel

**Affiliations:** 1 Institute of Human Genetics, University Medical Center Hamburg-Eppendorf, 20246 Hamburg, Germany; k.kirchner@uke.de (K.K.); b.sievers@uke.de (B.S.); 2 Department of Oncology, Hematology and Bone Marrow Transplantation with Division of Pneumology, University Medical Center Hamburg-Eppendorf, 20246 Hamburg, Germany; c.seidel@uke.de (C.S.); f.paulsen@uke.de (F.-O.P.); cbokemeyer@uke.de (C.B.); 3 Institute of Human Genetics, University Hospital Salzburg, Paracelsus Medical University, 5020 Salzburg, Austria

**Keywords:** testicular germ cell tumors, germ cell tumors, *CHEK2*

## Abstract

Testicular germ cell tumors (TGCTs) represent the most frequent malignancy in young adult men and have one the highest heritability rates among all cancers. A recent multicenter case–control study identified *CHEK2* as the first moderate-penetrance TGCT predisposition gene. Here, we analyzed *CHEK2* in 129 TGCT cases unselected for age of onset, histology, clinical outcome, and family history of any cancer, and the frequency of identified variants was compared to findings in 27,173 ancestry-matched cancer-free men. We identified four TGCT cases harboring a P/LP variant in *CHEK2* (4/129, 3.10%), which reached statistical significance (*p* = 0.0191; odds ratio (OR), 4.06; 95% CI, 1.59–10.54) as compared to the control group. Cases with P/LP variants in *CHEK2* developed TGCT almost 6 years earlier than individuals with *CHEK2* wild-type alleles (5.67 years; 29.5 vs. 35.17). No association was found between *CHEK2* status and further clinical and histopathological characteristics, including histological subtypes, the occurrence of aggressive TGCT, family history of TGCT, and family history of any cancer. In addition, we found significant enrichment for the low-penetrance *CHEK2* variant p.Ile157Thr (*p* = 0.0259; odds ratio (OR), 3.69; 95% CI, 1.45–9.55). Thus, we provide further independent evidence of *CHEK2* being a moderate-penetrance TGCT predisposition gene.

## 1. Introduction

Testicular germ cell tumor (TGCT) is the most frequent malignancy in young men aged 15–45 years. Based on histology, TGCTs are classically divided into seminoma and non-seminoma, the latter including choriocarcinomas, teratomas, and yolk sac and embryonal carcinomas. Moreover, in about 5% of individuals, germ cell tumors (GCTs) occur at extragonadal sites [1]. However, it is postulated that all GCTs likely arise from incompletely differentiated primordial germ cells (PGCs) [2]. This common etiology is further supported by both clinical findings, e.g., a lack of familial clustering of a specific histological subtype [3], as well as previous genetic data, which failed to identify a consistent difference in genotype frequencies for any histological subtype [4]. A large-scale integrative analysis of somatic alteration in these tumors established TGCTs to have a high aneuploidy and low point-mutation rate, followed by strong differences between histological subtypes concerning the global DNA methylation and microRNA expression [5].

Regarding the germline genetic basis, TGCTs are actually estimated to have the third highest heritability among all cancers [6], with an estimated heritability of 49% from familial correlations [7]. Genome-wide association studies (GWASs) have identified several strong hits in biologically plausible loci that may affect pathways that are thought to promote TGCT susceptibility like germ cell development, male primordial germ cells specification, sex determination and maturation, epigenetic reprogramming, apoptosis, and chromosomal segregation [8,9,10]. Intriguingly, despite these huge successes, for a long time, no Mendelian susceptibility genes, e.g., moderately or highly penetrant susceptibility variants, have been consistently linked to TGCTs.

Indeed, only recently, using different discovery strategies in three independent TGCT cohorts, a multicenter study has shown that pathogenic/likely pathogenic (P/LP) germline variants in *CHEK2*, encoding the checkpoint kinase 2 (CHK2), are strongly enriched in all three cohorts, thereby identifying *CHEK2* as the first TGCT moderate-penetrance susceptibility gene [11]. Namely, carriers of germline P/LP variants in *CHEK2* were 4–6 times more likely to develop TGCTs than unaffected male individuals and, on average, had a 6-year earlier age of presentation than TGCT men with wild-type *CHEK2* alleles. Moreover, the low-penetrance *CHEK2* variant (p.Ile157Thr) was found to be a Croatian founder TGCT risk variant [11].

To further validate these findings, we performed a comprehensive *CHEK2* analysis in 129 men of European ancestry with TGCTs, unselected for early onset or a positive family history of TGCTs, which were ascertained at the University Medical Center Hamburg-Eppendorf.

## 2. Materials and Methods

### 2.1. Study Participants

A total of 129 men of European ancestry with a primary germ cell tumor, 107 with a testicular germ cell tumor (TGCT) and 22 with an extragonadal germ cell tumor (EGCT) (Table S1), unselected for age of onset, histology, clinical outcome, and family history of TGCT and any cancer, were ascertained by the Department of Oncology, Hematology, and Bone Marrow Transplantation with the Division of Pneumology, University Medical Center Eppendorf, Hamburg, Germany. Patients were classified as having a less aggressive form of the disease if the cancer was categorized as stage I. In all other cases, the patients were classified as having a more aggressive form, as previously described [12]. Clinical data on all cases were uniformly obtained from oncology specialists using a structured questionnaire. Written informed consent was obtained from each individual after a detailed explanation of the purpose of the study. The study was performed in accordance with the Declaration of Helsinki protocols and performed in accordance with protocols approved by the Ethics Committee of the Hamburg Chamber of Physicians: PV 3802. As a control group, we used the cancer-free non-Finnish European individuals of the Exome Aggregation Consortium (ExAC) cohort, excluding the cases included in The Cancer Genome Atlas (TCGA) project [13], as previously described [11].

### 2.2. Genetic Analyses

The extraction of genomic DNA from whole-blood samples, PCR amplification, and the Sanger sequencing of all 14 coding exons of *CHEK2* were performed as previously described [11,14]. In addition, to the previously described protocols for the detection of exon 9_10 deletion [11,14], the occurrence of this additional deletion was verified by PCR amplification as described previously [15]. The clinical interpretation of identified *CHEK2* variants followed the American College of Medical Genetics and Genomics (ACMG) criteria, as previously described [11,14].

### 2.3. Statistical Analyses

For statistical analysis, Fischer’s exact test and an Unpaired *t*-test with Welch’s correction were performed using GraphPad Prism 8.

## 3. Results

### 3.1. Clinical Characteristics of the Studied Cases

A detailed clinical and pathological characterization of the 129 germ cell tumor (GCT) men included in the study is shown in Table S1. In summary, the mean age at the onset of GCTs was 34.99 years (ranging from 17 to 69 years). The gross majority, 107 cases (83%), developed a testicular germ cell tumor (TGCT), whereas 22 cases (17%) developed either a mediastinal or retroperitoneal extragonadal germ cell tumor (EGCT). Regarding the histology, 47 cases (36.4%) developed a seminoma and 82 cases (63.6%) developed a non-seminoma. Out of the 129 GCT cases included in this study, only two cases developed a second GCT: UKE-TKZT-26 developed an EGCT non-seminoma and UKE-TKZT-74 developed a TGCT seminoma. According to the staging, following recommendations by the American Joint Committee on Cancer (AJCC), the TNM Classification of Malignant Tumours (TNM), 44 cases (34%) had a stage I, 33 cases (25.7%) had a stage II, and 52 cases (40.3%) had a stage III GCT. 

Family history of GCT occurrence was available for 115 cases (89%), and only four cases (3.48%) reported a GCT in first- and second-degree relatives. Moreover, family history of any malignancy was available for only 92 cases (71.3%). Out of the latter, 58 cases (63%) reported at least one malignancy in first- and second-degree relatives (Table 1).

### 3.2. Identification of Rare CHEK2 Variants

**Figure 1:**
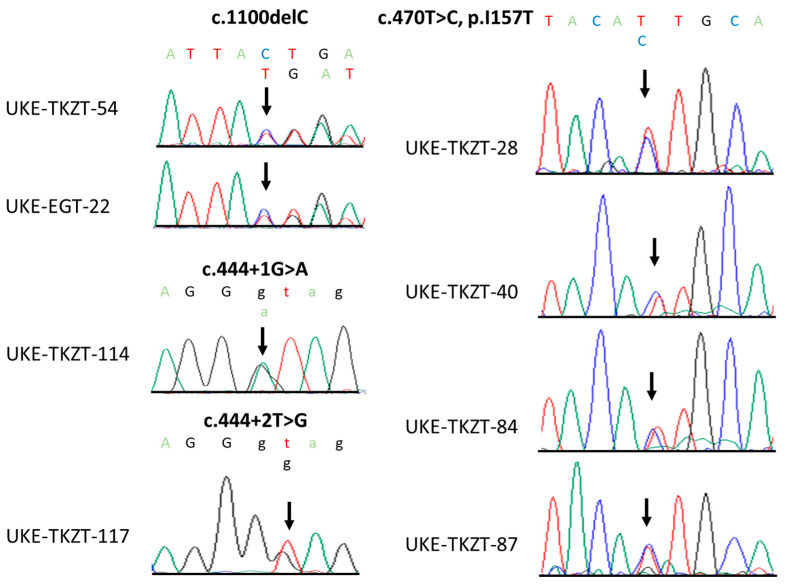
Sequence chromatograms of identified loss-of-function and p.Ile157Thr *CHEK2* variants. Shown are the positions of respective variants (black arrow).

**Figure 2:**
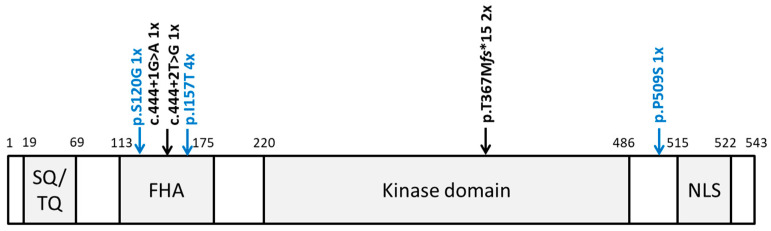
Location of all identified *CHEK2* variants. Schematic protein structure of CHK2 showing conserved domains. SQ/TQ: SQ/TQ cluster domain; FHA: forkhead-associated domain; kinase domain; NLS: nuclear localization signal. Pathogenic and likely pathogenic variants are shown in black above the protein structure; the other variants are shown in blue.

The direct Sanger sequencing of *CHEK2* followed by PCR amplification for the detection of exon 9_10 deletion identified altogether six heterozygous, non-synonymous variants. Two cases harbored the c.1100delC, p.Thr367Met*fs*15*, and one case harbored the c.444 + 1G > A (IVS2 + 1G > A). Notably, both of these variants have been previously identified in TGCT cases and classified as pathogenic [11]. Moreover, we identified one case harboring a novel variant, c.444 + 2T > G (IVS2 + 2T > G), which, similarly to the c.444 + 1G > A, affects the splice donor site in intron 2 (Figure 1 and Figure 2). Indeed, Splice Site Prediction by Neural Network (Berkeley Drosophila Genome Project; http://www.fruitfly.org/seq_tools/splice.html, accessed on 10 May 2023) [16] suggested that, similar to the c.444 + 1G > A, this variant also leads to an abnormal splicing and a 4-bp insertion. Thus, c.444 + 2T > G eliminates part of the forkhead-homology-associated domain and the entire kinase activation domain of CHK2 [17,18]. Taken together, we identified pathogenic or likely pathogenic (P/LP) loss-of-function (LOF) *CHEK2* germline variants in four GCT cases (4/129, 3.10%). Moreover, a comparison to the control group reached statistical significance, and the LOF germline *CHEK2* variant carriers were four times more likely to develop TGCTs than unaffected male individuals (*p* = 0.0191; odds ratio (OR), 4.06; 95% CI, 1.59–10.54; Table 2).

In addition, we identified four cases (4/129, 3.10%) harboring the low-penetrance variant c.470T > C, p.Ile157Thr (Figure 1 and Figure 2), which was previously suggested to be a Croatian founder TGCT risk variant [11]. Comparing these data to the control group, the difference also reached statistical significance (*p* = 0.0259; Table 2). Similar to the findings in the Croatian cohort [11], carriers of p.Ile157Thr were almost four times more likely to develop TGCTs than unaffected male individuals (odds ratio (OR), 3.69; 95% CI, 1.45–9.55; Table 2).

Additionally, we identified a single GCT case harboring c.358A > G, p.Ser120Gly, and a single GCT case harboring c.1525C > T, p.Pro509Ser. The p.Ser120Gly variant is located within the forkhead-associated domain (FHA) and has so far not been identified in large datasets, such as the Exome Aggregation Consortium (ExAC) [13] or the Genome Aggregation Database (gnomAD) [19]. However, due to the lack of confirmatory functional analysis and further affected individuals harboring this variant, following the American College of Medical Genetics and Genomics (ACMG) criteria, this variant was classified as a variant of unknown significance (VUS). The p.Pro509Ser variant, located between the kinase domain and the nuclear localization signal (NLS), was previously shown not to interfere with the CHK2 function [20] and was identified with a frequency of 0.0001 in gnomAD. Thus, according to the ACMG criteria, this variant was classified as likely benign (Figure 2).

### 3.3. Clinical and Histopathological Characteristics of CHEK2 Germline Variant Carriers Identified in This Study

To compare the clinical and histopathological characteristics of GCT cases in our study, stratified by *CHEK2* germline variant status, we next performed a case–case analysis. Carriers of *CHEK2* LOF variants developed TGCTs almost 6 years earlier than non-*CHEK2* LOF carriers (5.67 years; 29.5 vs. 35.17). However, this finding reached only borderline significance (*p* = 0.2315). Moreover, a further comparison regarding the histology, the occurrence of aggressive TGCTs, family history of TGCTs, and family history of any cancer all revealed statistically insignificant *p*-values (Table 3).

Similarly, a comparison of carriers of the low-penetrance variant p.Ile157Thr to the individuals with *CHEK2* wild-type alleles did not yield significant *p*-values for any of the tested clinical and histopathological characteristics (Table 3). It is worth noting that three out of four TGCT p.Ile157Thr carriers developed a seminoma, and all four had a stage > 1 TGCT. However, none of these findings met statistical significance, *p* = 0.1489 and *p* = 0.2995, respectively (Table 3).

### 3.4. Meta-Analysis of Histological Subtypes and Age of Diagnosis of CHEK2 Germline Variant Carriers Identified So Far

Next, in order to assess the potential clinical utility of *CHEK2* testing in TGCT cases, we compared the available clinicopathological findings in all TGCT carriers of LOF and p.Ile157Thr that have so far been documented in the literature. In more detail, we included 27 TGCT carriers of a LOF *CHEK2* variant and 52 TGCT carriers of the p.Ile157Thr *CHEK2* variant [11,21]. Unfortunately, only the data on the histological subtypes and the age of onset were available for all the cases. The youngest TGCT carrier of a *CHEK2* LOF variant was 18 years of age, whereas the oldest at diagnosis was 41 years of age [11]. The mean age of diagnosis was 28.19 years. Interestingly, the youngest TGCT carrier of a *CHEK2* p.Ile157Thr variant was even younger, 14 years of age [21], whereas the oldest at diagnosis was 51 years of age [11]. The mean age of diagnosis was 32.52 years. Further analysis, revealed no preference for a histological subtype in either TGCT *CHEK2* LOF or p.Ile157Thr carriers (Table 4). Thus, these data suggest that *CHEK2* genetic testing should be offered to all TGCT cases, regardless of age of diagnosis or histological subtype.

## 4. Discussion

Checkpoint kinase 2, a serine/threonine protein kinase encoded by *CHEK2*, is a well-established tumor suppressor [22]. Germline variants in *CHEK2* have been associated with increased susceptibility to various cancers with the most compelling evidence regarding breast, colorectal, and prostate cancers [17,23,24]. The latter is not unexpected given the broad role of CHK2 in the DNA damage response (DDR) pathway, including the sensing of DNA double-strand breaks, the regulation of the cell cycle, and the blockade of DNA replication [25]. Notably, CHK2 has additionally DDR-independent biological functions including the regulation of proper chromosome segregation during both meiosis [26] and mitosis [27], defects in which are the major cause of aneuploidy [28]. Notably, several studies suggested TGCTs to be characterized by marked aneuploidy rates [5,29,30]. Thus, *CHEK2* constituted a highly plausible TGCT candidate gene. Indeed, a recent multicenter case–control study identified *CHEK2* as the first moderate-penetrance predisposition gene, a finding that was later also replicated in the Russian population [21].

To corroborate these findings, we have ascertained a further cohort and carried out the first independent replication study in men with TGCTs of European ancestry who were unselected for age of onset, clinical outcome, family history of TGCTs, and family history of any cancer. Indeed, we identified pathogenic or likely pathogenic (P/LP) loss-of-function (LOF) *CHEK2* germline variants in 3.10% of unselected TGCT cases that were significantly enriched compared to the control group. Moreover, the LOF germline *CHEK2* variant carriers were four times more likely to develop TGCTs than unaffected, cancer-free male individuals of European ancestry (*p* = 0.0191; odds ratio (OR), 4.06; 95% CI, 1.59–10.54; Table 2). Notably, these data are strikingly similar to the previous findings in unselected TGCT men [11], strongly supporting *CHEK2* as a moderate-penetrance TGCT predisposition gene. Concerning our patient cohort, the majority presented with metastatic disease and non-seminomatous histology. Furthermore, with 17%, the fraction of extragonadal germ cell tumors is relatively high. This is comprehensible, as it can be assumed that the number of patients with advanced and/or refractory disease is higher in a referral center. However, as the patients detected with *CHEK2* germline variants are under current follow-up care after treatment, there is no evidence that this genetic alteration leads to a more aggressive course or chemotherapy refractoriness. 

Several large-scale analyses in various other cancers suggested a reduced penetrance of p.Ile157Thr as compared to the P/LP *CHEK2* variants [20,23,31]. Previous functional analyses have shown that p.Ile157Thr indeed affects the function of CHK2 by impairing binding to CDC25A [32] and by affecting its dimerization ability in a dominant negative manner, resulting in a lack of auto-phosphorylation [33]. Out of the four TGCT cohorts studied so far [11,21], p.Ile157Thr was previously associated with TGCT only in the cohort of Croatian men, which led to the hypothesis that it may represent a Croatian founder TGCT risk variant [11]. Here, we identified p.Ile157Thr in 3.10% of unselected TGCT cases of European ancestry, a finding that also reached statistical significance when compared to the control group, thus providing further evidence that p.Ile157Thr might be a TGCT low-penetrance variant.

Previous case–case analysis comparing the clinical and histopathological characteristics of TGCT cases, stratified by *CHEK2* variant status, suggested that P/LP *CHEK2* variant carriers have an earlier age of onset (with a mean of 5.95 years) and an increased risk of developing a contralateral TGCT as compared to *CHEK2* wild-type allele carriers [11]. Similarly, individuals with *CHEK2* LOF in this study also showed a trend, which did not, however, reach statistical significance, towards an earlier age of onset (5.67 years; 29.5 vs. 35.17; *p* = 0.2315; Table 3). It is worth noting that, with only two cases who developed a second TGCT, we were underpowered to analyze the possible association with an increased risk of developing a second TGCT with the *CHEK2* variant status. Furthermore, similar to the previous study [11], statistically insignificant *p*-values were obtained after a further comparison regarding histology, the occurrence of aggressive TGCTs, family history of GCTs, and family history of any cancer. In addition, and contrary to the previous study [11], the p.Ile157Thr carriers included in this study did not show a trend regarding the earlier age of onset. However, we did observe a trend, which did not reach statistical significance, towards an association with seminomas and aggressive TGCTs. Clearly, much larger studies are needed to further delineate these putative associations.

The genetic basis of TGCTs remains extremely complex, both in terms of various somatic alterations and their high heritability. Indeed, multi-omics molecular characterization of somatic alterations, including analyses on genomic, epigenetic, transcriptomic, and proteomic levels, has only recently identified histology-specific differences in microRNA expression and global DNA methylation patterns [5]. Likewise, despite several genetic approaches to identify moderate- to high-penetrance TGCT susceptibility genes [34,35,36], *CHEK2* remains the only moderate-penetrance TGCT predisposition gene [11,37]. In addition, a recent gene-burden analysis, utilizing whole-exome sequencing (WES) in high-risk TGCT cases and bilateral and familial cases, independently confirmed the previously established association between *CHEK2* and TGCT predisposition in their dataset [11,38]. Furthermore, the latter study identified associations of TGCTs with LOF variants in 10 genes and nonsynonymous variants in 41 genes. Although the identified genes represent biologically plausible candidate genes, larger studies are needed to confirm these findings and to elucidate whether these genes represent low- or moderate-penetrance TGCT genes. Moreover, the latter study further suggested that, unlike the situation in many other tumor types, there does not seem to be a single major, highly penetrant TGCT gene [38]. Thus, current data suggest that the high heritability is primarily due to a polygenic etiology [38,39]. It is, however, tempting to speculate that at least a small part of the TGCT heritability might be due to ultra-rare variants in high-penetrant genes. Further studies aiming to identify high-penetrant TGCT variants may take advantage of the fact that biological samples of the parents might be available, due to the young age of the cases, and thus perform WES analyses in a trio setting. Indeed, motivated by the identification of the first high-penetrance gene for hepatocellular carcinoma [40], our group is currently conducting similar analyses in high-risk TGCT cases. 

Moreover, similarly challenging remains the treatment of platinum-refractory TGCTs [41,42]. Hence, further delineation of the genetic basis of TGCTs, with special emphasis on the identification of novel moderate- to high-penetrance TGCT genes, along with putative germline variants that may influence therapeutic approaches, will be the major focus of our future studies.

Last but not least, the sample size of our cohort and, accordingly, the usage of a well-established large ExAC dataset are clear limitations of our study. For this reason, we additionally compared the clinicopathological characteristics of all TGCT *CHEK2* LOF and p.Ile157Thr variant carriers documented so far in the literature [11,21]. Unfortunately, only the age of diagnosis and histological subtype were available for all men. We did not observe a preference concerning a specific histological subtype. Furthermore, although cases with *CHEK2* LOF developed TGCTs almost 6 years earlier, given that the oldest case was 41 at diagnosis, we suggest that the age of diagnosis cannot be taken as an inclusion criterion for *CHEK2* genetic testing. Importantly, since *CHEK2* variant carriers might additionally have an increased risk of developing further malignancies [17,24], we suggest that germline *CHEK2* testing should be offered to all TGCT cases. Indeed, these men might profit from early screening for prostate and colorectal cancer. Germline-directed treatment in terms of poly (ADP-ribose) polymerase (PARP) inhibitors might in the future be an additional utility for germline *CHEK2* genetic testing in TGCT cases, as is already established for metastatic castration-resistant prostate cancer [43].

## 5. Conclusions

Taken together, our study provides further evidence of *CHEK2* as a moderate-penetrance TGCT susceptibility gene associated with an early age of onset. In addition, our data suggest that the low-penetrance *CHEK2* variant, p.Ile157Thr, leads to increased TGCT risk beyond the Croatian population. In addition, we suggest that all men with a TGCT should be offered a germline *CHEK2* analysis.

## Figures and Tables

**Table 1:** Clinicopathological characteristics of the 129 patients included in this study.

Status Total	Cases *n* = 129
Mean age at diagnosis (range)	34.99 (17–69)
Anatomical site	
Testicular (%)	107 (83)
Extragonadal (%)	22 (17)
Histology	
Seminoma (%)	47 (36.4)
Non-seminoma (%)	82 (63.6)
Stage grouping (AJCC)	
Stage I (%)	44 (34)
Stage II (%)	33 (25.7)
Stage III (%)	52 (40.3)
Family history of GCT	
(%; available for cases)	4 (3.48; 115)
Family history of any tumor	
(%; available for cases)	58 (63; 92)

**Table 2:** Enrichment analysis: frequency of *CHEK2* variants identified in German GCT patients compared to cancer-free men of European ancestry.

*CHEK2* Variants	Individuals, No. (%)		OR (95% CI) Per Allele	*p*-Value
	Controls	Cases		
	(*n* = 27,173)	(*n* = 129)		
LOFs	210 (0.8)	4 (3.1)	4.06 (1.59–10.54)	**0.0191**
c.470T > C, p.I157T	231 (0.9)	4 (3.1)	3.69 (1.45–9.55)	**0.0259**

LOFs, loss-of-function variants (c.1100delC, p.T367Mfs*15; c.444 + 1G > A; c.444 + 2T > G). Significant *p*-values are depicted in bold.

**Table 3:** Clinicopathological characteristics of carriers of *CHEK2* variants and non-carriers.

	*CHEK2* Non LOF Carriers	*CHEK2* LOF Carriers	*p*-Value	*CHEK2* WT	*CHEK2* p.I157T Carriers	*p*-Value
Age of diagnosis (mean) ^a^	35.17	29.5	0.2315	35.14	36	0.9022
Family history of GCT ^b^	4/111 (111/125 patients)	0/4	>0.9999	4/109 (109/121 patients)	0/4	>0.9999
Family history of any cancer ^b^	55/88 (88/125 patients)	3/4	>0.9999	53/86 (86/121 patients)	2/2	0.5259
Histology (seminoma vs. non-seminoma) ^b^	45/80	2/2	0.6219	44/77	3/1	0.1489
Aggressive GCT (>stage I) ^b^	83/125	2/4	0.6053	79/121	4/4	0.2995

^a^ Unpaired *t*-test with Welch’s correction; ^b^ Fischer’s exact test.

**Table 4:** Meta-analysis of TGCT carriers of *CHEK2* variants.

	*CHEK2* Non-LOF Carriers	*CHEK2* p.I157T Carriers
Age of diagnosis (mean)	18–41 (28.19)	14–51 (32.52)
Histology (seminoma vs. non-seminoma)	14/13	26/26

## Data Availability

Data are contained within the article and Supplementary Materials.

## Supplementary Materials

The following supporting information can be downloaded at: https://www.mdpi.com/article/10.3390/jcm12227065/s1, Table S1: Clinical, histopathological, and genetic characteristics of the cohort.
